# Impacts of climate change on labor productivity: a narrative review

**DOI:** 10.47626/1679-4435-2025-1524

**Published:** 2025-12-09

**Authors:** Bruna Roberta Muntanelli, Felipe Seiti Sekiya, Evangelina da Motta Pacheco Alves de Araujo, Alberto José Niituma Ogata, João Silvestre Silva-Junior

**Affiliations:** 1 Hospital das Clínicas, Faculdade de Medicina da Universidade de São Paulo (HCFMUSP), São Paulo, SP, Brazil; 2 São Paulo School of Business Administration, Fundação Getulio Vargas (FGV EAESP), São Paulo, SP, Brazil; 3 Department of Legal Medicine, Bioethics, Occupational Medicine, and Physical Medicine and Rehabilitation, FMUSP, São Paulo, SP, Brazil; 4 Department of Medicine, Centro Universitário São Camilo, São Paulo, SP, Brazil

**Keywords:** climate change, occupational health, environmental health., | mudança climática, saúde ocupacional, saúde ambiental.

## Abstract

Climate change, characterized by long-term shifts in global weather patterns, exerts significant impacts on ecosystems and human health. This study aimed to assess the influence of climate change and natural disasters on labor productivity. A literature review was conducted in PubMed, including studies investigating exposure to climate change or natural disasters and their relationship with productivity. Of the 774 records initially identified, 12 studies were eligible for analysis. All assessed heat overload associated with global warming. Heat stress was found to be related to substantial productivity losses in labor-intensive outdoor activities, particularly in agriculture and construction, with estimated reductions of up to 80% in vulnerable regions - such as Southeast Asia, Latin America, and Sub-Saharan Africa - and under more pessimistic warming scenarios. Evidence suggests that adaptation measures, such as adjusting work schedules, can partially mitigate these effects, although with limited effectiveness in severe warming contexts. Climate change compromises labor productivity, with heat stress as a central factor. Although workplace adaptations are necessary, they are insufficient under extreme scenarios. The findings highlight the importance of investment in climate mitigation and research, with special attention to vulnerable regions such as Brazil, and the expansion of research scope to include other outcomes, such as air pollution, increased vector-borne diseases, and intensified extreme weather events.

## Abstract (pt)

As mudanças climáticas, caracterizadas por alterações de longo prazo nos padrões climáticos globais, exercem impactos relevantes sobre ecossistemas e saúde humana. Este estudo teve como objetivo analisar a influência das mudanças climáticas e de desastres naturais na produtividade laboral. Realizou-se revisão bibliográfica na base PubMed, incluindo estudos que investigaram a exposição a mudanças climáticas ou a desastres naturais e sua relação com produtividade. Dos 774 registros inicialmente identificados, 12 estudos foram elegíveis para análise. Todos avaliaram a sobrecarga térmica associada ao aquecimento global. O estresse térmico mostrou-se relacionado a perdas substanciais de produtividade em atividades laborais intensivas ao ar livre, especialmente na agricultura e na construção civil, com estimativas de redução de até 80% em regiões vulneráveis - como Sudeste Asiático, América Latina e África Subsaariana - e em cenários mais pessimistas de aquecimento. As evidências indicam que medidas de adaptação, como a readequação de horários de trabalho, podem mitigar parcialmente esses efeitos, mas com efetividade limitada em contextos de aquecimento severo. Conclui-se que as mudanças climáticas comprometem a produtividade laboral, tendo o estresse térmico como fator central. Embora adaptações no ambiente laboral sejam necessárias, mostram-se insuficientes em cenários extremos. Reforça-se a importância de investimentos em mitigação climática e em pesquisa, com atenção especial a regiões vulneráveis, como o Brasil, e a ampliação do escopo para outros desfechos, incluindo poluição do ar, aumento de doenças transmitidas por vetores e intensificação de eventos climáticos extremos.

## INTRODUCTION

According to the Climate Dictionary of the United Nations Development Programme, climate change refers to long-term transformations in the Earth’s climate that warm the atmosphere, oceans, and land surfaces, alter ecosystems, threaten biodiversity, and affect human health. These changes also intensify extreme events - such as hurricanes, floods, heat waves, and droughts - while ocean warming and polar ice loss raise sea levels and accelerate coastal erosion, resulting in severe consequences for several regions.^1^

At the same time, the United Nations General Assembly, one of the main bodies of the United Nations (UN), defines natural disasters as serious disruptions in the functioning of communities or societies, arising from the interaction between hazardous events, vulnerable conditions, and limited response capacity, with potential human, economic, and environmental losses that often require external intervention.^2^

The Lancet Countdown South America, a collaboration among 21 academic institutions and UN agencies, analyzed the relationships between public health and climate change in South American countries. The 2022 edition covered 12 countries and provided evidence for adaptation and mitigation policies. In Brazil, the impacts on health and productivity were notable: in 2021, losses of approximately US$ 11.2 billion were estimated due to reduced work capacity associated with high temperatures, particularly in the construction and agricultural sectors. Between 2000 and 2021, about 62.8 million hectares of forest cover were lost, mainly from deforestation linked to agricultural expansion, which hindered mitigation and increased exposure to pollutants and food insecurity. Although initiatives such as the national assessment of climate vulnerability in health existed, challenges in human and financial resources persisted.^3^

A review of 170 studies identified consistent effects of climate change on occupational health and safety, including heat-related illnesses, thermal stress, higher accident rates, kidney disease, mortality, and decreased productivity.^4^

Labor productivity is classically defined as the relationship between the resources employed (inputs) and the results obtained (outputs) in a production system.^5^ Changes in workers’ health negatively affect productivity through absenteeism (absence from work) and presenteeism (reduced performance at work).^6^

Although references exist on the impacts of climate change on productivity, the topic still lacks in-depth analysis. Therefore, this study aims to identify and synthesize evidence on how climate change and natural disasters affect workers’ productivity, contributing to filling gaps in the literature.

## METHODS

This narrative review used a bibliographic search of articles published in scientific journals indexed in the PubMed database. The search was conducted on October 16, 2024, using the following strategy with descriptors and Boolean operators: (climate change OR natural disasters) AND workers productivity.

Studies addressing exposure to climate change and/or natural disasters and reporting occupational health outcomes related to productivity were included. Review articles, intervention studies, study protocols, and research that did not assess the exposure or outcomes of interest were excluded. Records without full-text access and publications in languages other than English, Spanish, or Portuguese were also excluded.

## RESULTS

A total of 774 records were identified through the bibliographic search. After screening titles and abstracts, 94 articles were selected for full-text review; the remaining records were excluded for not addressing the topics of interest - climate change and/or natural disasters as exposure and labor productivity as the outcome. During the full-text review, 85 articles were excluded, mostly because they examined climate change and extreme events without directly measuring productivity.

**Figure 1:**
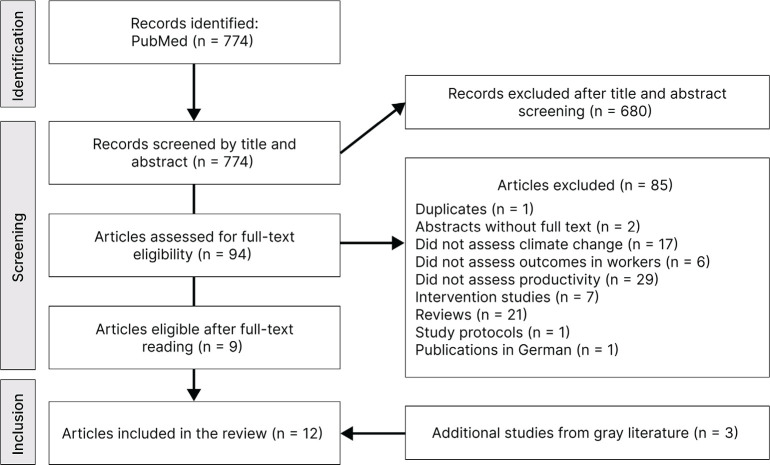
Preferred Reporting Items for Systematic Reviews and Meta-Analyses flowchart of study search via database.

In the end, nine studies were eligible for detailed analysis, to which three records from the gray literature were added, totaling 12 studies, as shown in the Preferred Reporting Items for Systematic Reviews and Meta-Analyses flowchart (Figure 1). The analyzed populations included workers from diverse geographic contexts - India, Indonesia, Türkiye, China, European countries, Central America, and Southeast Asia - with no studies conducted in Brazil or focused on the Brazilian population.

The methodological designs included cross-sectional observational studies (samples between 124 and 286 workers),^7^,^8^ one field trial (361 workers),^9^ regional or global climate modeling studies,^10^-^17^ and multi-model analyses,^18^ which made it possible to examine a range of scenarios and estimate the impacts of climate change on labor productivity.

### ASSESSMENT OF CLIMATE CHANGE

The included studies assessed exposure to heat overload using different approaches to project the impacts of rising temperatures on work environments and conditions. The main strategies were: use of the Wet Bulb Globe Temperature (WBGT) Index^7^,^10^-^18^; climate simulations with regional models-such as the Regional Climate Model, version 3 (RegCM3)^12^ - or global models^10^,^13^-^15^,^17^ based on the Special Report on Emissions Scenarios (SRES) and the Representative Concentration Pathways (RCPs); and analyses of historical climate series.^11^,^16^

The WBGT, employed in multiple studies,^7^,^10^-^18^ integrates air temperature, humidity, solar radiation, and wind speed, and is widely used to estimate heat effects in occupational settings. This metric is particularly relevant in sectors with direct environmental exposure, such as agriculture and construction, and is central to quantifying levels of heat overload and their effects on health and productivity.

RegCM3 was used to anticipate impacts at the regional scale,^12^ incorporating local features - relief, land use, and ecosystems - to refine projections of how warming may affect specific areas and their working populations.

Global climate models,^9^,^13^-^15^,^17^ in turn, cover continental to planetary scales and support climate-change projections based on emissions scenarios. The SRES, developed by the Intergovernmental Panel on Climate Change (IPCC) in 2000, explore alternative socioeconomic trajectories through the end of the 21st century (for example, A1, with high globalization and rapid economic growth; and B2, oriented toward sustainability and clean technologies). The RCPs, introduced later, represent radiative forcing trajectories - from RCP2.6 (low emissions, consistent with limiting global warming to < 2 °C) to RCP8.5 (high emissions, without substantive control)-offering a standardized framework to assess risks and to plan mitigation and adaptation with a focus on protecting workers and productivity.

### ASSESSMENT OF LABOR PRODUCTIVITY

Productivity was measured using multiple approaches: individual or aggregate production volume^7^,^17^; the High Occupational Temperature Health and Productivity Suppression (HOTHAPS) questionnaire^7^,^8^; projections that relate work intensity and rest fractions to the WBGT^10^,^11^,^15^,^16^,^18^; estimates of work hours lost associated with heat stress^13^,^14^,^17^; and the decreased labor productivity (DLP) formula proposed by Altinsoy & Yildirim^12^ for evaluating productivity losses at work.

HOTHAPS collects information on working conditions, heat exposure, breaks, work pace, and symptoms of heat stress (eg, fatigue and dehydration),^19^ and also allows assessment of the effectiveness of heat-adaptation measures.

Productivity estimates based on WBGT use the index to determine the capacity to maintain output under intense heat, defining the percentage of work capacity after adjustments for breaks recommended for thermal recovery; productivity declines as the frequency and duration of these breaks increase.

The work-hours-lost assessment method estimates the impact of adverse conditions - most notably heat overload - on the hours actually worked, by calculating the difference between planned and completed hours, considering breaks, reduced pace, and heat-related absences.

The formula proposed by Altinsoy & Yildirim^12^ quantifies productivity losses as workdays lost due to excessive heat. It considers temperature ranges (27.5-36 °C) and the rest percentages associated with each range, allowing estimation of break time or inability to work.

No study assessed the relationship between natural disasters and labor productivity. Chart 1 presents the included articles.

**Chart 1:** Description of articles selected for review

Year	Authors	Study design	Population	Location	Environmental variable (exposure)	Exposure measure	Outcome measure (productivity)
2010	Kjellstrom et al.^10^	Climate modeling	Agricultural, industrial, and service workers	Global	Heat stress related to climate change	Global climate models (SRES) + WBGT	Work capacity as a function of WBGT
2013	Kjellstrom et al.^11^	Climate modeling	Heat-exposed workers	Southeast Asia (Brunei Darussalam, Cambodia, Indonesia, Laos, Malaysia, Myanmar, Philippines, Singapore, Thailand, Vietnam)	Heat stress related to climate change	Climate data + WBGT trends (HOTHAPS soft) + WBGT	Work capacity as a function of WBGT
2013	Sahu et al.^7^	Cross-sectional observational	Rice harvest workers (n = 124)	West Bengal (India)	Heat stress related to climate change	WBGT calculation + WBGT trends (HOTHAPS soft)	Worker output/hour + HOTHAPS questionnaire
2015	Altinsoy & Yildirim^12^	Climate modeling	Manual workers, mainly outdoor (agriculture and construction)	Western Türkiye	Heat stress related to climate change	Regional climate model RegCM3 + WBGT	Formula for productivity loss at work
2017	Hooyberghs et al.^13^	Climate modeling	Office workers in urban areas	Europe (Antwerp, Bilbao, London)	Indoor heat increase due to climate change	Global climate models (RCPs) + WBGT	Work-hour losses as a function of WBGT
2018	Kjellstrom et al.^14^	Climate modeling	Shaded workers, moderate activity	Global	Indoor heat increase due to climate change	Global climate models (RCPs) + WBGT	Work-hour losses as a function of WBGT
2018	Takakura et al.^15^	Climate modeling	Outdoor workers, various regions	Global	Indoor heat increase due to climate change	Global climate models (RCPs) + WBGT	Work capacity as a function of WBGT
2019	Pogačar et al.^8^	Cross-sectional observational	Outdoor workers (n = 286)	Greece and Slovenia	Heat stress related to climate change	Heatwave (national meteorological services definition)	HOTHAPS questionnaire
2021	Masuda et al.^9^	Field trial	Rural community workers (n = 361)	East Kalimantan (Indonesia)	Loss of tropical forests with increased heat exposure	Field experiment (forest loss/heat) + WBGT	Worker output per minute and break behavior
2021	Dasgupta et al.^18^	Empirical multi-model study	Indoor and outdoor workers, various regions	Global	Heat stress related to climate change	Multi-model climate projections + WBGT	Work capacity as a function of WBGT and effective work (labor supply × productivity)
2021	Parsons et al.^16^	Climate modeling	Heavy manual workers (agriculture, fishing, forestry, construction)	Global	Heat stress related to climate change	Climate reanalysis + climate projections + WBGT	Work capacity as a function of WBGT
2023	Cheng et al.^17^	Climate modeling	Workers from multiple sectors	China	Heat stress related to climate change	Global climate models (RCPs) + WBGT	Work-hour losses as a function of WBGT

HOTHAPS = High Occupational Temperature Health and Productivity Suppression; RCPs = Representative Concentration Pathways; RegCM3 = Regional Climate Model, version 3; SRES = Special Report on Emissions Scenarios; WBGT = Wet Bulb Globe Temperature.

## DISCUSSION

The results were organized according to study design, with brief context in each topic. There was consensus on the association between different manifestations of climate change and reduced labor productivity.

### CLIMATE MODELING STUDIES

Climate modeling simulates future scenarios and their potential impacts based on historical data (temperature, precipitation, and other variables), integrated by algorithms that project changes in working conditions. The studies by Kjellstrom et al.^10^,^11^,^14^ offer a comprehensive analysis of occupational heat and productivity.

The initial study^9^, conducted in 2009, employed SRES scenarios to project productivity losses up to 2080 in various regions: in the A2 scenario (high emissions), estimates ranged from 11% to 27%, especially in tropical and subtropical areas; in the B2 scenario (lower emissions), losses were more moderate, up to 16%, particularly in outdoor activities without thermal control. In 2013, focusing on Southeast Asia, the authors used maps of temperature, humidity, and WBGT to project losses through 2050: up to 80% in heavy work and 50% in moderate work with direct sun exposure.^11^

In 2018, they assessed work-hour losses in tropical and subtropical regions^13^: above 35 °C, strenuous activities suffer substantial reductions; losses currently < 2% may exceed 6% under 1.5 °C warming (RCP2.6) and reach 12%-16% at 2.7 °C (RCP6.0), with regions such as India and Cambodia reaching approximately 11% of annual hours compromised.

Taken together, the three studies converge on the same conclusion: adopting adaptation measures and mitigation policies is imperative to curb the effects of global warming on productivity. In their absence, heat overload will progressively restrict work capacity - especially in tropical and subtropical regions, where outdoor activities predominate and thermal control is limited.^10^,^11^,^14^

Hooyberghs et al.^13^ investigated the impact of heat overload on the productivity of office workers in Antwerp, Bilbao, and London. Between 1986 and 2005, work-hour losses in Antwerp ranged from 1% in north-facing rooms to over 4% in south-facing rooms, highlighting the effect of solar orientation. Changing work hours reduced work-hour losses to under 2% in south-facing rooms, while measures such as increased ventilation and installation of solar blinds decreased work-hour losses to 0.8% and 0.12%, respectively. Owing to its lower latitude, Bilbao showed higher work-hour losses. Projections indicate that, absent interventions, these losses could quadruple by 2100. The study concludes that structural measures - such as increased ventilation and the use of solar blinds - are more effective for heat mitigation than simply changing work schedules.

Takakura et al.^15^ examined the effects of heat stress on outdoor activities, aggravated by climate change. In the reference period, average work capacity was 82%, with 18% of time devoted to preventive breaks. In the RCP2.6 scenario, with temperature increase limited to 2 °C, projected capacity for 2090 is 76%, requiring an average 1.1-hour shift earlier in the start of the workday. In the high-emissions RCP8.5 scenario, capacity falls to 54%, with 46% of time reserved for breaks and start-time shifts of up to 5.7 hours. Under this condition, about half of workers would need to begin their shift before 3:00 a.m.

In high-latitude regions, the impact is minimal because temperatures do not reach critical levels. By contrast, in low-latitude areas, the required shift can reach 7.5 hours under RCP8.5. Although artificial lighting can bring forward start times, sunrise is a practical limit. Under such conditions, many places would require work to start before dawn, and shifts under 3 hours would not offset productivity losses without stricter climate mitigation. Thus, economic losses cannot be avoided solely through this adaptation.^15^

Parsons et al.^16^ evaluated productivity losses associated with heat overload and observed that they increase exponentially with temperature: under 1% at a WBGT of 20 °C, around 10% at 27 °C, 50% at 32.5 °C, and up to 90% at 38 °C. In low-latitude cities such as New Delhi and Doha, workers lose 15-20 minutes per hour at noon, whereas in the early morning hours losses are under 10 minutes. Shifting work to cooler periods partially reduces the impact, but its effectiveness diminishes as global temperature rises.

Taken together, the three studies reinforce that reorganizing work schedules - concentrating activities during the cooler hours of the day - can alleviate productivity losses. However, all point to significant limitations of this strategy under more severe warming scenarios.^13^,^15^,^16^

Regional studies corroborate this trend, indicating that heat overload intensified by climate change significantly reduces labor productivity. In Türkiye, Altinsoy & Yildirim^12^ estimated substantial losses in the agricultural and construction sectors, especially during summer. The additional rest time required increased after 2040, resulting in productivity drops of 39% in 2041-2070 and 48% in 2071-2100. In agriculture, losses were projected to be 5%-8%, aligning with forecasts of sharp declines in tropical regions.

Complementarily, Cheng et al.^17^, in China, projected substantial losses of annual work hours across climate scenarios. Global warming could increase lost hours by up to 121.1% by the end of the century compared with the current scenario. However, limiting warming to 1.5 °C would avoid about 53.9% of these losses. These findings highlight the urgency of climate-mitigation and adaptation policies, especially in sectors with high heat exposure and in warmer-climate regions.

### EMPIRICAL MULTI-MODEL STUDY

The multi-model study by Dasgupta et al.^18^ evaluated, on a global scale, the combined effects of climate change on productivity and labor supply. In regions with WBGT > 25 °C, average labor productivity under low heat exposure decreases by 8%, potentially reaching 64% in areas near the Equator. The most affected regions are South America, Central Africa, India, and Southeast Asia. Under the same low-exposure conditions, global effective work - defined as the relationship between labor supply and productivity - is expected to decline by 6.7% under 1.5 °C warming, 10.3% under 2.0 °C, and 18.3% under 3.0 °C. Africa and Asia would experience the steepest declines - 25.9% and 18%, respectively - in the 3.0 °C scenario; under high exposure and 3.0 °C warming, average projected reductions in effective work would be 32.8% in Africa and 25.1% in Asia. These results reinforce the need for adaptation measures to mitigate the effects of heat overload on workers’ health and the economy, especially in vulnerable areas.

### CROSS-SECTIONAL OBSERVATIONAL STUDIES

Sahu et al.^7^ investigated 124 rice-harvest workers in India and found that productivity decreases by approximately 5% for every 1 °C increase in WBGT above 26 °C. In addition, 40% reported productivity loss on the hottest days, suggesting that heat stress may reduce future productive capacity, particularly in tropical settings.

In a study conducted in Greece and Slovenia, Pogačar et al.^8^ reported that 69% of Greek workers and 71% of Slovenian workers who operate outdoors perceived heat stress as significantly affecting productivity. Approximately 24% of Greek participants and 16% of Slovenian participants reported losses exceeding 30% during heatwaves.

Together, these findings indicate that heat stress substantially impairs productivity - especially in outdoor activities and warmer climates - and highlight the importance of preventive measures and educational actions to protect health and maintain work capacity.

### FIELD TRIAL STUDIES

In a field trial conducted in Indonesia, Masuda et al.^9^ observed an 8.22% reduction in labor productivity associated with a local temperature increase of up to 2.84 °C due to deforestation. The study identified more frequent heat-related breaks as the main behavioral mechanism adopted by workers and found that increased financial incentives did not elevate productivity. These findings reinforce the need for effective adaptive strategies to mitigate the impacts of rising temperatures.

Climate change directly affects productivity, mainly due to rising temperatures and increased frequency of heatwaves, with the most intense impacts occurring in outdoor activities in tropical and subtropical regions. Projections indicate that, in the absence of adaptation and emissions control, regions such as Southeast Asia, Africa, and Latin America may face substantial losses by the end of the 21st century. The effects of heat are particularly severe in heavy labor, where sun exposure reduces work capacity and imposes more frequent and extended breaks; even in moderate activities, high temperatures decrease efficiency and pose risks to health and safety.

According to the 2024 report of the Lancet Countdown, in 2023 the potential loss of global income associated with reduced work capacity due to extreme heat reached US$ 835 billion (0.82% of the global economy). Average losses were more pronounced in countries with low and medium human development index (HDI), equivalent to 7.6% and 4.4% of gross domestic product, respectively. It was also estimated that 512 billion potential work hours were lost in 2023, with the greatest burden in lowand middle-HDI countries (averages of 221 and 291 hours lost per worker). Low-HDI nations - responsible for 71% of global hours lost - were especially affected in the agricultural sector, which accounted for 63% of losses.^20^

Adaptation measures such as adjusting work schedules to cooler periods and providing heat-protective infrastructure are recommended, but their effectiveness diminishes as global temperatures rise. This scenario underscores the urgency of climate action to limit warming, or else work capacity in vulnerable regions will be sharply compromised, with significant consequences for health, the economy, and global productivity.

Regarding knowledge gaps, the systematic review by Ferrari et al.^4^ indicates that the literature is predominantly concentrated on temperature rise and occupational health outcomes. There is a need to broaden the analysis to include other climate-change effects, such as air pollution, vector-borne diseases, and extreme weather events. Additionally, more studies have focused on outdoor workers - particularly in agriculture and construction. A complementary scoping review in Australia emphasized the importance of assessing productivity in indoor environments.^21^ Meanwhile, the review by Borg et al.^22^ revealed a lack of information on how factors such as age, gender, business size, and occupational sector jointly influence productivity - gaps that represent opportunities for future research to yield more specific and precise estimates in the context of climate change.

Finally, no investigations were identified that specifically assessed the effects of natural disasters on the productivity of affected workers. Theoretically, events such as hurricanes, floods, droughts, and wildfires may substantially reduce productivity by affecting labor availability and work capacity, interrupting productive activities, causing equipment losses, and limiting access to workplaces, in addition to affecting physical and mental health. The combination of these effects compromises both individual performance and overall economic productivity, especially in regions with a high recurrence of disasters.

## CONCLUSIONS

Multiple approaches can measure labor productivity, depending on the type of activity and the purpose of the assessment. Among objective methods, highlights include counts of units produced or tasks completed and the output-to-input ratio, which are widely used in production settings. In this review, only two studies employed objective metrics (eg, bundles of rice and stacks of corn sacks per unit time), demonstrating the relationship between productivity and environmental exposure.

Other strategies include sectoror role-specific key performance indicators - such as sales targets, service time, error rates, and efficiency - as well as self-report instruments that capture perceived limitations at work, such as the Work Limitations Questionnaire^23^,^24^ and the Stanford Presenteeism Scale.^25^,^26^ Each method has advantages and limitations; therefore, combinations of quantitative and qualitative approaches (including peer interviews) are often recommended for more comprehensive evaluations.

Although Brazil is highly vulnerable to the effects of climate change - including natural disasters that may reduce productive capacity - no specific studies were found that estimate such impacts using field data in the country. It is crucial to develop research that validates, in the Brazilian context, the international consensus on productivity declines under warming scenarios and that identifies effective, feasible interventions. Evidence of this nature is essential to guide public policies aligned with sustainable development and with the protection of workers’ health.

## References

[1] United Nations Development Programme (UNDP) (2023). The climate dictionary - Speak climate fluently.

[2] United Nations (2016). Report of the open-ended intergovernmental expert working group on
indicators and terminology relating to disaster risk reduction.

[3] Hartinger SM, Yglesias-González M, Blanco-Villafuerte L, Palmeiro-Silva YK, Lescano AG, Stewart-Ibarra A (2023). The 2022 South America report of The Lancet Countdown on health
and climate change: trust the science. Now that we know, we must
act. Lancet Reg Health Am.

[4] Ferrari GN, Leal GCL, Thom de Souza RC, Galdamez EVC (2023). Impact of climate change on occupational health and safety: a
review of methodological approaches. Work.

[5] Mendes R, Machado Junior JA (2018). Dicionário de saúde e segurança do trabalhador:
conceitos, definições, história, cultura.

[6] Beaton D, Bombardier C, Escorpizo R, Zhang W, Lacaille D, Boonen A (2009). Measuring worker productivity: frameworks and
measures. J Rheumatol.

[7] Sahu S, Sett M, Kjellstrom T. (2013). Heat exposure, cardiovascular stress and work productivity in
rice harvesters in India: implications for a climate change
future. Ind Health.

[8] Pogačar T, Žnidaršič Z, Kajfež Bogataj L, Flouris AD, Poulianiti K, Črepinšek Z. (2019). Heat waves occurrence and outdoor workers’ self-assessment of
heat stress in Slovenia and Greece. Int J Environ Res Public Health.

[9] Masuda YJ, Garg T, Anggraeni I, Ebi K, Krenz J, Game ET (2021). Warming from tropical deforestation reduces worker productivity
in rural communities. Nat Commun.

[10] Kjellstrom T, Kovats RS, Lloyd SJ, Holt T, Tol RS. (2009). The direct impact of climate change on regional labor
productivity. Arch Environ Occup Health.

[11] Kjellstrom T, Lemke B, Otto M. (2013). Mapping occupational heat exposure and effects in South-East
Asia: ongoing time trends 1980-2011 and future estimates to
2050. Ind Health.

[12] Altinsoy H, Yildirim HA. (2015). Labor productivity losses over western Turkey in the twenty-first
century as a result of alteration in WBGT. Int J Biometeorol.

[13] Hooyberghs H, Verbeke S, Lauwaet D, Costa H, Floater G, De Ridder K. (2017). Influence of climate change on summer cooling costs and heat
stress in urban office buildings. Clim Change.

[14] Kjellstrom T, Freyberg C, Lemke B, Otto M, Briggs D. (2018). Estimating population heat exposure and impacts on working people
in conjunction with climate change. Int J Biometeorol.

[15] Takakura J, Fujimori S, Takahashi K, Hasegawa T, Honda Y, Hanasaki N (2018). Limited role of working time shift in offsetting the increasing
occupational-health cost of heat exposure. Earth’s Future.

[16] Parsons LA, Shindell D, Tigchelaar M, Zhang Y, Spector JT. (2021). Increased labor losses and decreased adaptation potential in a
warmer world. Nat Commun.

[17] Cheng L, Gu K, Zhao L, Wang H, Ji JS, Liu Z (2023). Projecting future labor losses due to heat stress in China under
climate change scenarios. Sci Bull (Beijing).

[18] Dasgupta S, van Maanen N, Gosling SN, Piontek F, Otto C, Schleussner CF. (2021). Effects of climate change on combined labour productivity and
supply: an empirical, multi-model study. Lancet Planet Health.

[19] Climate CHIP. Hothaps protocol.

[20] Romanello M, Walawender M, Hsu SC, Moskeland A, Palmeiro-Silva Y, Scamman D (2024). The 2024 report of the Lancet Countdown on health and climate
change: facing record-breaking threats from delayed action. Lancet.

[21] Wuersch L, Neher A, Marino FE, Bamberry L, Pope R. (2023). Impacts of climate change on work health and safety in Australia:
a scoping literature review. Int J Environ Res Public Health.

[22] Borg MA, Xiang J, Anikeeva O, Pisaniello D, Hansen A, Zander K (2021). Occupational heat stress and economic burden: a review of global
evidence. Environ Res.

[23] Lerner D, Amick BC (2001). 3rd, Rogers WH, Malspeis S, Bungay K, Cynn D. The work
limitations questionnaire. Med Care.

[24] Soárez PC, Kowalski CC, Ferraz MB, Ciconelli RM. (2007). Translation into Brazilian Portuguese and validation of the work
limitations questionnaire. Rev Panam Salud Publica.

[25] Koopman C, Pelletier KR, Murray JF, Sharda CE, Berger ML, Turpin RS (2002). Stanford presenteeism scale: health status and employee
productivity. J Occup Environ Med.

[26] Paschoalin HC, Griep RH, Lisboa MT, Mello DC. (2013). Transcultural adaptation and validation of the Stanford
Presenteeism Scale for the evaluation of presenteeism for Brazilian
Portuguese. Rev Lat Am Enfermagem.

